# The effects of e‐cigarette use on asthma severity in adult BALB/c mice

**DOI:** 10.1113/EP092959

**Published:** 2025-09-14

**Authors:** Jenield J. D'abreo, Emily K. Chivers, Peter B. Noble, Luke J. Berry, Rachel R. Huxley, Arthur (Bill) W. Musk, Peter J. Franklin, Benjamin J. Mullins, Katherine R. Landwehr, Alexander N. Larcombe

**Affiliations:** ^1^ Respiratory Environmental Health, Wal‐yan Respiratory Research Centre The Kids Research Institute Australia Nedlands WA Australia; ^2^ School of Human Sciences The University of Western Australia Perth WA Australia; ^3^ The George Institute for Global Health University of New South Wales Barangaroo NSW Australia; ^4^ Faculty of Health Deakin University Burwood Victoria Australia; ^5^ School of Population and Global Health The University of Western Australia Perth WA Australia; ^6^ Occupation, Environment and Safety, School of Population Health Curtin University Perth WA Australia

**Keywords:** asthma severity, electronic cigarette, inflammation, lung function, mouse

## Abstract

Electronic cigarettes (e‐cigarettes) are often perceived to be a less harmful alternative to tobacco cigarettes. Potentially due to this perception, they are used by people with pre‐existing respiratory conditions, such as asthma, who otherwise would not smoke. Despite this, there are few studies exploring the health effects of e‐cigarette use on pre‐existing asthma. In this study, a house dust mite‐induced allergic‐airways disease phenotype was generated in adult BALB/c mice over 7 weeks. For the last 2 weeks of this period, mice were also exposed to either medical air, or tobacco smoke or e‐cigarette aerosol (with or without nicotine) for 2 h/day. Twenty‐four hours later, respiratory parameters including lung volume/function and responsiveness to methacholine were assessed. Biological samples were taken for analysis of pulmonary cellular inflammation and mediator levels, serum IgE and lung/airway structure. There were complex effects of exposure on respiratory outcomes. For example, tobacco smoke‐exposed mice of both sexes were the most responsive to methacholine but had suppressed total cellular and eosinophilic inflammation. Female e‐cigarette aerosol‐exposed mice had impaired parenchymal mechanics at functional residual capacity compared with tobacco smoke‐exposed mice, irrespective of nicotine. Interferon γ levels were suppressed in both e‐cigarette‐exposed groups. There was no effect of any exposure on IgE or lung structural parameters. E‐cigarette aerosol exposure exacerbated aspects of an allergic airways disease phenotype in mice. This suggests that asthmatics should exercise increased caution if thinking of using e‐cigarettes.

## Abstract (highlights)

- **What is the central question of this study?**
  Does e‐cigarette use lead to increased asthma severity and/or lead to asthma exacerbations in a mouse model of house dust mite‐induced allergic airways disease?
- **What is the main finding and its importance?**
  We found that e‐cigarette aerosol exposure exacerbated some aspects of the allergic airways disease phenotype in mice, suggesting that asthma sufferers should exercise caution if thinking about vaping.

## 1 INTRODUCTION

Electronic cigarettes (e‐cigarettes) are devices that heat and aerosolise a flavoured liquid (primarily consisting of humectants such as propylene glycol and glycerine), which may or may not contain nicotine, producing an aerosol for inhalation (Grana et al., 2014). In recent years e‐cigarette use has increased (Carroll Chapman & Wu, 2014; Shannon et al., 2014; Wilson & Wang, 2017), particularly in adolescents and young adults (Hennekens et al., 2024; Tehrani et al., 2022). This may be partly due to the perception that they are a less harmful alternative to tobacco cigarettes (Gorukanti et al., 2017; Romijnders et al., 2018), or even that they are completely safe to use (Larcombe et al., 2023). However, e‐cigarettes have only been in widespread use for a relatively short period of time (Versella & Leyro, 2019), and data on their long‐term health effects are scarce (Banks et al., 2023). This is important with respect to the potential health effects of e‐cigarette use in individuals with existing respiratory diseases such as asthma (Clapp & Jaspers, 2017; Fedele et al., 2016).

Asthma is a chronic respiratory condition, with a pathophysiology characterised by airway inflammation and bronchial hyper‐responsiveness upon provocation, which leads to reversible airway obstruction (Fireman, 2003). Asthma symptoms can be exacerbated by environmental pollutants, including tobacco smoke (Hamid & Tulic, 2009), which is a key reason that some scientists and physicians advocate for asthmatic smokers to switch to e‐cigarettes (Polosa et al., 2014; Polosa, Campagna et al., 2016, 2016). Importantly, e‐cigarette use has been associated with asthma and asthma‐like symptoms in numerous population‐based studies (Bhatta & Glantz, 2020; Cho & Paik, 2016; Osei et al., 2019) and there is an increased risk of asthma exacerbations following second‐hand e‐cigarette aerosol exposure (Bayly et al., 2019). Adolescents with asthma are also more likely to use e‐cigarettes (and smoke cigarettes) compared with their non‐asthmatic peers (Ringlever et al., 2013; Schweitzer et al., 2017).

E‐cigarette aerosol exposure can impact lung function, lung inflammation and immune responses to pulmonary infection in humans (reviewed in Hickman & Jaspers, 2020), all of which are important in an asthma context; however, data are sometimes contradictory. For example, Lappas et al. (2018) showed that after just 5 min of e‐cigarette use, both ‘healthy’ and mild asthmatic smokers (*n* = 27 per treatment) exhibited increased respiratory system impedance and resistance, and decreased fractional exhaled nitric oxide (FeNO) (Lappas et al., 2018). Conversely, another study found that 1 h of exposure to nicotine‐free and flavour‐free e‐cigarette aerosol did not impact lung function in asthmatic individuals (Boulay et al., 2017), although some participants reported cough and difficulty breathing along with mucus secretions. This suggests that e‐cigarette excipients alone could impact respiratory function, a finding which is supported by short‐term exposure studies (in non‐asthmatics) involving propylene glycol mist/theatrical fog (Varughese et al., 2005; Wieslander et al., 2001) where exposure resulted in increased throat symptoms (e.g. cough, upper airway irritation) and slight decreases in lung function. Similarly, there is also uncertainty with respect to the effects of e‐cigarette aerosol exposure, nicotine and flavouring chemicals on asthma in animal‐based studies (Chapman et al., 2019; Song et al., 2023; Taha et al., 2020). For example, some studies show an effect of nicotine on certain markers of an asthma‐like phenotype (e.g. interleukin (IL)‐13, neutrophilia, mucous) in asthmatic mice, but not non‐asthmatic mice (Song et al., 2023; Taha et al., 2020), while others show significant impacts due to flavouring chemicals and a suppression of pulmonary inflammation after e‐cigarette exposure in asthmatic mice (Chapman et al., 2019).

Despite the epidemiological associations between e‐cigarette use and asthma, the complex and contradictory findings from exposure studies indicate that a mechanistic basis for e‐cigarette use to increase asthma severity and/or lead to exacerbations requires additional research. Therefore, this study aimed to explore the effects of flavoured e‐cigarette use (with and without nicotine) on asthma severity using a house dust mite‐induced allergic airways disease murine model, and to compare these effects with both clean air and cigarette smoke exposure. We hypothesised that e‐cigarette aerosol exposure would exacerbate key features of asthma, such as airway hyper‐responsiveness and inflammation, but that these effects would be less severe than tobacco smoke exposure‐induced impacts.

## 2 METHODS

### 2.1 Ethical approval

All the procedures conducted in this study were approved by the Telethon Kids Institute Animal Ethics Committee (Approval No. 323) and were in accordance with the Australian *Code and Care and Use of Animals for Scientific Purposes* (8th ed., 2013). Our research complies with *Experimental Physiology*’s policies regarding animal experiments.

### 2.2 Animals and treatment groups

A total of 96 adult BALB/c mice (48 males and 48 females) aged 7 weeks were obtained from the Animal Resources Centre (Murdoch, WA, Australia). Mice were housed in individually ventilated cages (Sealsafe, Tecniplast, Buguggiate, Italy) on non‐allergic dust‐free bedding (Shepards Specialty Papers, Chicago, IL, USA). Allergen‐free food (Specialty Feeds, Glen Forrest, WA, Australia) and water were provided ad libitum. The mice were kept on a 12 h light:dark cycle. Upon arrival, mice were randomly allocated to treatment groups; air control (Air), cigarette smoke (Smoke), e‐cigarette aerosol with nicotine (Nic) and e‐cigarette aerosol without nicotine (No‐Nic). We commenced the experiment with *n* = 12 per treatment per sex; however, prior to data acquisition we had to euthanise one male Nic mouse and one female No‐Nic mouse for issues not related to the experimental protocol.

### 2.3 House dust mite exposure

Mice were anaesthetised with inhaled isoflurane (<3% in air) (Somnosuite, Kent Scientific, Torrington, CT, USA) and inoculated intranasally with 25 µg of *Dermatophagoides pteronyssinus* protein (house dust mite (HDM): 18.2% w/w protein, 11400 Endotoxin Units (EU); Greer Laboratories, Lenoir, NC, USA) which had been dissolved in 50 µL saline. Drops of solution were pipetted onto the nostrils until inhaled. All the mice underwent this process 5 days a week for 7 weeks (total of 35 inoculations per mouse).

### 2.4 Air, cigarette smoke and e‐cigarette aerosol exposure protocol

All mice were whole body exposed in 27‐L exposure chambers (30 cm × 30 cm × 30 cm) as previously described (Larcombe et al., 2017). Briefly, using separate chambers for each treatment, which were thoroughly cleaned between exposures, mice were exposed for 2 h/day (1 h at 09.00 h and 1 h at 14.00 h), 5 days/week for the last 2 weeks of HDM exposure.

### 2.5 Air exposure

Medical air was delivered to the chamber via a regulated source at a rate of 3 L/min.

### 2.6 Smoke exposure

A cigarette smoking machine (inExpose; SCIREQ, Montreal, Canada) was used to deliver smoke to the chamber as previously described (Larcombe, Foong, Berry et al., 2011). We used Winfield Red cigarettes (≤16 mg of tar, ≤1.2 mg of nicotine; Philip Morris, Melbourne, Australia) and a 35 mL puff was delivered into the chamber per minute. Three cigarettes were used in the morning and three in the afternoon. Each cigarette was puffed ∼6–8 times and during each hour‐long session a new cigarette was used every 20 min thereby allowing the chamber to flush out with clean air between cigarettes. Throughout exposure medical air was delivered to the chamber at a rate of 3 L/min.

### 2.7 E‐cigarette aerosol exposure

E‐liquids were aerosolised using the Innokin iTaste MVP 4.0 vape mod (Innokin Technology, Shenzhen, China) with 0.28 Ω coils set to 100 W. Nicotine‐free e‐liquids consisting of a 50:50 propylene glycol and glycerin excipient were purchased (Central Vapors, McKinney, TX, USA) with laboratory grade nicotine (Sigma‐Aldrich, Castle Hill, NSW, Australia) added to achieve a concentration of 19.7 ± 0.7 mg/mL (confirmed via LCMS). We used a 50:50 mixture of two flavours (‘Butterscotch Tobacco’ and ‘Papa Smurf’ (fruity)) to encompass the range of most commonly used flavours. E‐cigarette aerosol (35 mL puff volume) was delivered manually into the chamber once per minute using a 50 mL syringe and a three‐way tap. Simultaneously, medical air was delivered to the chamber at 3 L/min.

### 2.8 Animal preparation

The above exposures were staggered across treatments/individuals so that all mice were studied 48 h after their final HDM inoculation and ∼16 h after their final cigarette/e‐cigarette exposure. The timing was based on previous literature showing that the peak of HDM‐induced allergic response occurred 48 h after the final inoculation (Luebke et al., 2001). Mice were prepared for lung function assessment as previously described (Larcombe et al., 2008). Briefly, they were anesthetised by intraperitoneal injection of 0.1 mL/10 g bodyweight of a solution containing ketamine (40 mg/mL) and xylazine (2 mg/mL) (Troy Laboratories, Glendenning, NSW, Australia). Approximately one‐third was given to induce surgical anaesthesia, one‐third was administered once the mouse was being mechanically ventilated, and one‐third given at the conclusion of lung function measurement to euthanise the mice prior to biological sample collection.

### 2.9 Lung mechanics and responsiveness to methacholine

Anaesthetised and tracheostomised mice were attached to a ventilator (Legacy *f*lexiVent; Scireq, Montreal, QC, Canada) via an endotracheal cannula and ventilated at 450 breaths/min with 8 mL/kg tidal volume and 2 cmH_2_O positive end expiratory pressure (Larcombe, Foong, Bozanich et al., 2011). After f5 min the lung volume history was standardised by three deep inflations to a transrespiratory pressure (*P*_rs_) of 20 cmH_2_O. The first of these three deep inspirations was used to calculate *V*_max_ (lung volume at *P*_rs_ = 20 cmH_2_O), compliance, specific compliance and %V10 (lung volume at *P*_rs_ = 10 cmH_2_O/lung volume at *P*_rs_ = 20 cmH_2_O) (Limjunyawong et al., 2015). Respiratory impedance (*Z*_rs_) was measured at functional residual capacity (FRC) and was partitioned into airway (R_aw_) and parenchymal (G and H) loads using the Constant Phase Model (Hantos et al., 1992). *Z*_rs_ measurements were made once per minute for 5 min at FRC and again once per minute for 5 min after mice were administered each of nebulised saline and increasing concentrations of methacholine (MCh; 0.1, 0.3, 1, 3, 10 and 30 mg/mL; acetyl β‐methacholine chloride, Sigma‐Aldrich, St Louis, MO, USA) via an ultrasonic nebuliser (DeVilbiss UltraNeb, Somerset, PA, USA). The highest values recorded at each dose were used for analyses. Sensitivity to MCh (the dose required for airway resistance, tissue damping and tissue elastance to increase 50% above the measurement made after the saline aerosol) was also calculated.

### 2.10 Assessment of cellular inflammation and mediators

After lung function measurement, 0.5 mL of saline was washed in and out of the lungs three times through the tracheal cannula to collect bronchoalveolar lavage (BAL) fluid. BAL samples were processed for total and differential cell counts as previously described (Larcombe, Foong, Bozanich et al., 2011). Supernatant mediator levels were assessed using the Bio‐Plex Pro Mouse Cytokine 23‐Plex assay (Bio‐Rad Laboratories, Hercules, CA, USA) according to the manufacturer's instructions.

### 2.11 Serum IgE

Blood samples were collected from each mouse via cardiac puncture, centrifuged and levels of total serum IgE were measured via ELISA (MabTech, Preston, Victoria, Australia).

### 2.12 Histology and morphometry

After lung function measurement and BAL collection, lungs were inflation‐fixed in situ at 10 cmH_2_O using 4% formaldehyde. Fixed lungs were embedded in paraffin wax and the left lung sectioned with a thickness of 5 µm at intervals of 500 µm along the proximal lung. Sections were then stained with Masson's trichrome, and stereological software was used to analyse the area of airway smooth muscle (ASM) and epithelial layers (newCAST; Visiopharm, Hørsholm, Denmark) as well as the basement membrane internal perimeter (*P*_bm_). In order to account for the variance in airway size, the square root of these areas were normalised to *P*_bm_ (James et al., 1988).

### 2.13 Statistics

Data were analysed using GraphPad Prism (Version 5.02, GraphPad Software, San Diego, CA, USA) and SigmaPlot v14.0 (v14 Systat Software, Chicago, IL, USA). Two‐way ANOVA with the Holm‐Šidák *post hoc* test was used to analyse all data, with sex and treatment as factors. Data were transformed where required to satisfy the assumptions of normality and equal variance and are presented as means ± standard deviation. *P* < 0.05 was considered statistically significant.

## 3 RESULTS

### 3.1 Weight

At the commencement of study, there was no difference in weight between male mice (*P* = 0.656) or female mice (*P* = 0.089) randomly allocated to the different treatments (Table 1). On the day of lung function assessment/biological sample collection there was no effect of treatment on the weights of male mice (*P* = 0.105); however, female mice exposed to cigarette smoke were significantly lighter than female mice of every other treatment (*P* < 0.009 in all cases). There was a trend in both sexes for Air mice to be the heaviest, followed by the two e‐cigarette aerosol‐exposed treatments, with Smoke mice being the lightest.

**TABLE 1:** Weights of male and female BALB/c mice with house‐dust‐mite induced allergic airways disease and exposed to Air, e‐cigarette aerosol with and without nicotine (Nic and No‐Nic, respectively) or cigarette smoke (Smoke) at the commencement and conclusion of the study.

Treatment	Male		Female	
	Start (day 1)	End (day 35)	Start (day 1)	End (day 35)
Air	20.75 ± 0.95	24.00 ± 1.08	17.08 ± 1.71	19.31 ± 1.17
No‐Nic	20.83 ± 0.65	23.45 ± 0.85	16.73 ± 0.91	19.23 ± 1.46
Nic	21.01 ± 0.82	23.58 ± 1.31	16.81 ± 1.88	19.16 ± 1.56
Smoke	20.58 ± 0.85	22.91 ± 0.96	15.59 ± 0.88	17.38 ± 0.88^a^

Values are means ± SD (*n *= 11–12 per group) in grams (g). ^a^Significant difference between Smoke females and all other female treatments at the same time point.

### 3.2 Lung function at functional residual capacity

There were significant overall effects of both sex and treatment on airway resistance, tissue damping and tissue elastance measured at functional residual capacity (*P* < 0.004 in all cases; Table 2). Female mice had higher R_aw_, G and H at FRC than male mice regardless of treatment (*P* < 0.004 in all cases). Within sex, female Smoke mice had significantly lower R_aw_ and G compared with Nic or No‐Nic females (*P* < 0.028 in all cases), and they also had lower H compared with Nic females (*P* = 0.015). There were no other significant differences for female mice. Male Smoke mice had significantly lower R_aw_ than male Air mice (*P* = 0.045), and significantly lower H compared with male No‐Nic mice (*P* = 0.031).

**TABLE 2:** Lung function at functional residual capacity of male and female BALB/c mice with house dust mite‐induced allergic airways disease and exposed to Air, e‐cigarette aerosol with and without nicotine (Nic and No‐Nic, respectively) or cigarette smoke (Smoke).

Treatment	Male			Female		
	R_aw_ (cmH_2_O s mL^−1^)	G (cmH_2_O mL^−1^)	H (cmH_2_O mL^−1^)	R_aw_ (cmH_2_O s mL^−1^)	G (cmH_2_O mL^−1^)	H (cmH_2_O mL^−1^)
Air	0.36 ± 0.04^b^	5.41 ± 0.90	31.01 ± 5.76	0.36 ± 0.03	6.08 ± 0.73	34.53 ± 6.01
No‐Nic	0.35 ± 0.05	5.42 ± 0.88	32.85 ± 6.49^b^	0.39 ± 0.04^b^	7.21 ± 1.89^b^	37.18 ± 7.22
Nic	0.34 ± 0.03	5.05 ± 0.43	29.60 ± 2.54	0.38 ± 0.03^b^	6.74 ± 1.15^b^	38.76 ± 6.81^b^
Smoke	0.32 ± 0.03^a^	4.61 ± 0.63	26.90 ± 3.46	0.33 ± 0.04	5.51 ± 0.79	30.95 ± 5.49

Values are means ± SD (*n* = 11–12 per group).

^a^ Significant difference from Air within sex for a particular parameter.

^b^ Significant difference from Smoke within sex for a particular parameter.

### 3.3 Compliance

There was no effect of sex (*P* = 0.466) or treatment (*P* = 0.427) on specific compliance (Table 3). There were significant effects of both treatment (*P* < 0.001) and sex (*P* < 0.001) for *V*_max_ (lung volume at *P*_rs_ = 20 cmH_2_O). Males had a higher *V*_max_ regardless of treatment. Within sex, Smoke‐exposed mice had higher *V*_max_ compared with No‐Nic‐exposed mice (*P* < 0.033 in both cases), and Smoke females also had significantly higher *V*_max_ than female Nic (*P* < 0.001). There were also significant effects of both sex (*P* < 0.001) and treatment (*P* = 0.004) on compliance. Smoke‐exposed mice had higher compliance regardless of sex, and males had higher compliance regardless of treatment. There was a significant effect of treatment on %V10, with Smoke mice having lower %V10 than all other treatments regardless of sex (*P* < 0.022 in all cases). Within sex, female Smoke mice had lower %V10 than female Air and female Nic (*P* < 0.026 in both cases), while male Smoke mice had lower %V10 than male Air mice (*P* = 0.026).

**TABLE 3:** Lung volume at 20 cmH_2_O (*V*_max_), compliance (C), specific compliance (Cs) and %V10 of male and female BALB/c mice with house‐dust‐mite induced allergic airways disease and exposed to Air, e‐cigarette aerosol with and without nicotine (Nic and No‐Nic, respectively) or cigarette smoke (Smoke).

Treatment	Male				Female			
	*V*_max_ (mL)	C (mL/cmH_2_O^−1^)	Cs (cmH_2_O^−1^)	%V10	*V*_max_ (mL)	C (mL/cmH_2_O^−1^)	Cs (cmH_2_O^−1^)	%V10
Air	0.477 ± 0.074	0.043 ± 0.007	0.415 ± 0.153	81.9 ± 3.4	0.416 ± 0.055	0.039 ± 0.007	0.426 ± 0.159	81.7 ± 3.0^b^
No‐Nic	0.457 ± 0.094^b^	0.042 ± 0.009	0.412 ± 0.070	81.0 ± 2.1	0.410 ± 0.060^b^	0.039 ± 0.008	0.484 ± 0.113	80.2 ± 1.0
Nic	0.489 ± 0.051	0.046 ± 0.004	0.470 ± 0.109	80.1 ± 2.0	0.350 ± 0.078^b^	0.032 ± 0.009^b^	0.417 ± 0.087	81.2 ± 3.0^b^
Smoke	0.542 ± 0.069	0.049 ± 0.07	0.460 ± 0.109	78.7 ± 1.9^a^	0.492 ± 0.087	0.045 ± 0.009	0.493 ± 0.087	78.2 ± 3.0^a^

Values are means ± SD (*n* = 11–12 per group). ^a^Significant difference to Air within sex for a particular parameter. ^b^Significant difference to Smoke within sex for a particular parameter.

### 3.4 Responsiveness to methacholine and evocative concentration

**FIGURE 1:**
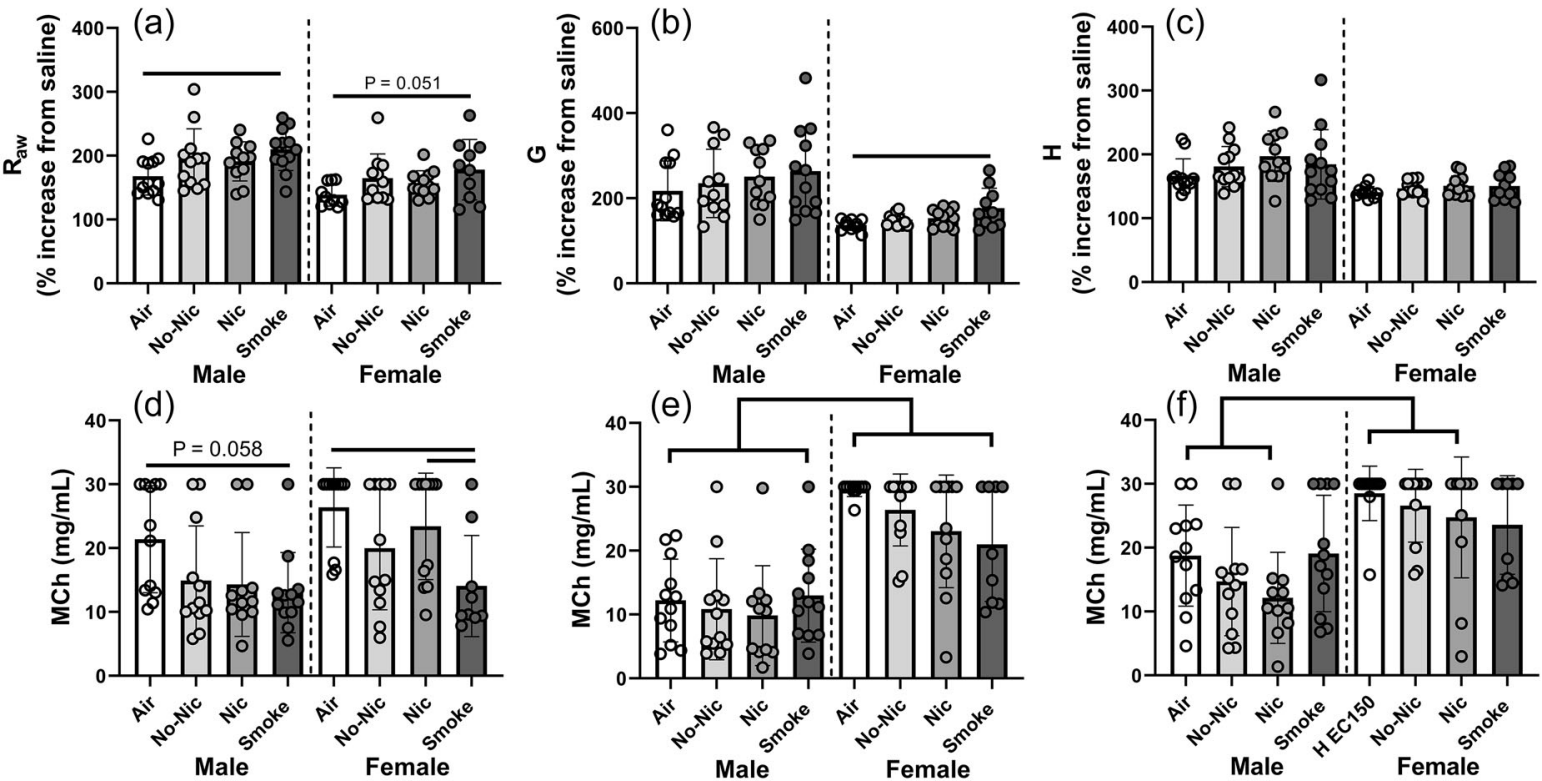
Percentage increase in response to methacholine for airway resistance (a), tissue damping (b) and tissue elastance (c), and the dose of methacholine required for a 50% increase from the value post‐saline aerosol for airway resistance (d), tissue damping (e) and tissue elastance (f) for male and female BALB/c mice with house dust mite‐induced allergic airways disease and exposed to Air, e‐cigarette aerosol with and without nicotine (Nic and No‐Nic, respectively) or cigarette smoke (Smoke). Note different scales. Circles represent individual mice (*n* = 11–12 per sex per treatment). Bars connect treatments that are significantly different from each other (*P* < 0.05).

There were significant effects of both treatment and sex on responsiveness to MCh (Figure 1). For percentage increase in airway resistance (i.e. percentage change between R_aw_ after the saline aerosol compared with R_aw_ after the 30 mg/mL MCh aerosol), there was a significant effect of sex (*P* < 0.001) and treatment (*P* = 0.002), but no interaction (*P* = 0.993; Figure 1a). Male mice were significantly more responsive regardless of treatment (*P* < 0.042 in all cases). Within sex, male Smoke mice were more responsive than male Air mice (*P* = 0.024), and female Smoke mice were borderline more responsive than female Air mice (*P* = 0.051); however, there were no other significant differences (*P* > 0.248 in all cases). Similar effects were seen in G, whereby there was a significant effect of sex (*P* < 0.001) and treatment (*P* = 0.035) but no interaction (*P* = 0.557; Figure 1b). Again, male mice were more responsive than females regardless of treatment (*P* < 0.002 in all cases). There was no effect of treatment within male mice (*P* > 0.745 in all cases), although female Smoke mice were significantly more responsive than female Air mice (*P* = 0.031) for this parameter. For H (Figure 1c), there was no effect of treatment (*P* = 0.169); however, male mice were more responsive than females regardless of treatment (*P* < 0.019 in all cases).

There were significant effects of sex (*P* < 0.001) and treatment (*P* = 0.004) on REC_150_ (the dose of MCh required to elicit a 50% increase in airway resistance; Figure 1d). Males were more responsive than females regardless of treatment (*P* = 0.004), although there was no effect of treatment within male mice (*P* > 0.058 in all cases). For female mice, those exposed to Smoke were more responsive than both Air and Nic mice (*P* < 0.044 in both cases). There was a similar significant effect of treatment on GEC_150_, whereby males were more responsive than females regardless of treatment (*P* < 0.001; Figure 1e). There was no effect of treatment on this parameter (*P* = 0.156). Conversely, there was no effect of sex on HEC_150_ (*P* = 0.159), but there was an overall effect of treatment (*P* < 0.001) with males exposed to air, Nic or No‐Nic being more responsive than females treated the same way (*P* < 0.003 in all cases; Figure 1f). There was no difference between male and female Smoke mice (*P* = 0.190).

### 3.5 Cellular inflammation

**FIGURE 2:**
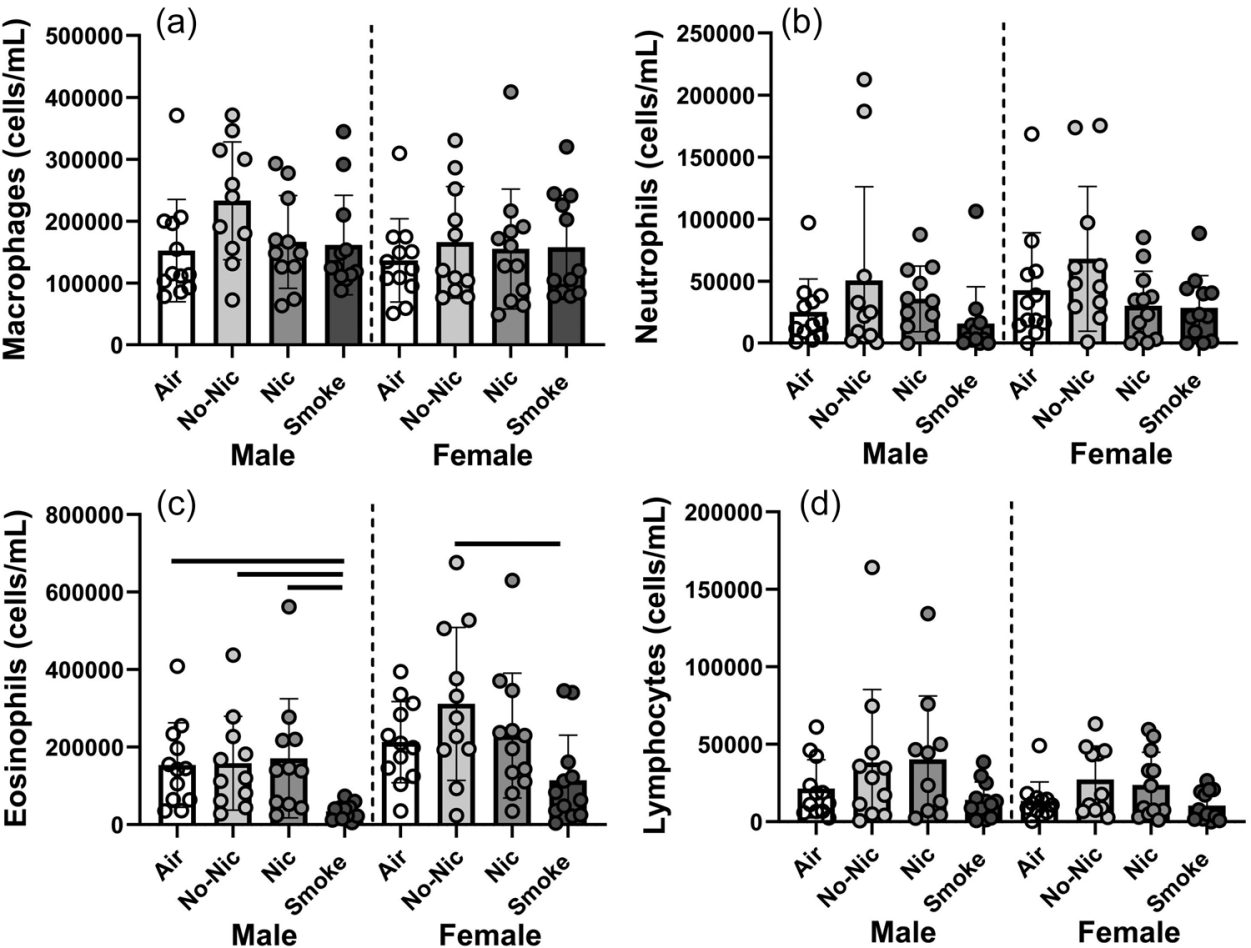
Cellular inflammation in BAL in terms of macrophages (a), neutrophils (b), eosinophils (c) and lymphocytes (d) for male and female BALB/c mice with house dust mite‐induced allergic airways disease and exposed to Air, e‐cigarette aerosol with and without nicotine (Nic and No‐Nic, respectively) or cigarette smoke (Smoke). Note different scales. Circles represent individual mice (*n* = 11–12 per sex per treatment). Bars connect treatments that are significantly different from each other (*P* < 0.05).

There was a significant effect of treatment on the total number of cells in BAL (*P* < 0.001; data not shown). Overall, smoke mice had significantly fewer total cells in their BAL than Nic or No‐Nic mice (*P* < 0.016 in both cases). Within sex, male smoke mice had significantly fewer total cells than male No‐Nic mice (*P* = 0.009). This difference was primarily due to differences in numbers of eosinophils (Figure 2c) and to a lesser extent differences in neutrophils (Figure 2b) and lymphocytes (Figure 2d) as there was no effect of treatment on macrophage numbers (*P* = 0.226). Smoke‐exposed mice had fewer neutrophils (*P* = 0.025) and lymphocytes (*P* = 0.050) than No‐Nic mice. With respect to eosinophils, there were significant effects of sex (*P* < 0.001) and treatment (*P* < 0.001). Smoke mice had fewer eosinophils than all other treatments for males (*P* < 0.011) and No‐Nic females (*P* = 0.003).

### 3.6 Serum IgE

There was no significant effect of sex (*P* = 0.464) or treatment (*P* = 0.204) on levels of serum IgE. For both sexes, serum IgE of Smoke mice (57.3 ± 34.4 ng/mL for females, and 49.7 ± 31.0 ng/mL for males) was considerably lower than that in any other treatment (averaged 107.0 ± 82.6 ng/mL for females and 99.1 ± 87.5 ng/mL for males), but this was not statistically significant.

### 3.7 Bronchoalveolar lavage mediators

Of the 23 mediators assessed in the kit, three (IL‐1β, IL‐12(p70) and granulocyte–macrophage colony‐stimulating factor (GM‐CSF)) were not detected above the limit of detection in any sample. Another six (IL‐3, IL‐6, IL‐9, IL‐10, IL‐13, granulocyte colony‐stimulating factor (G‐CSF)) were only detected in a small proportion of samples at very low levels. These mediators are excluded from analyses. For simplicity, significant effects of treatment are only reported within sex.

**FIGURE 3:**
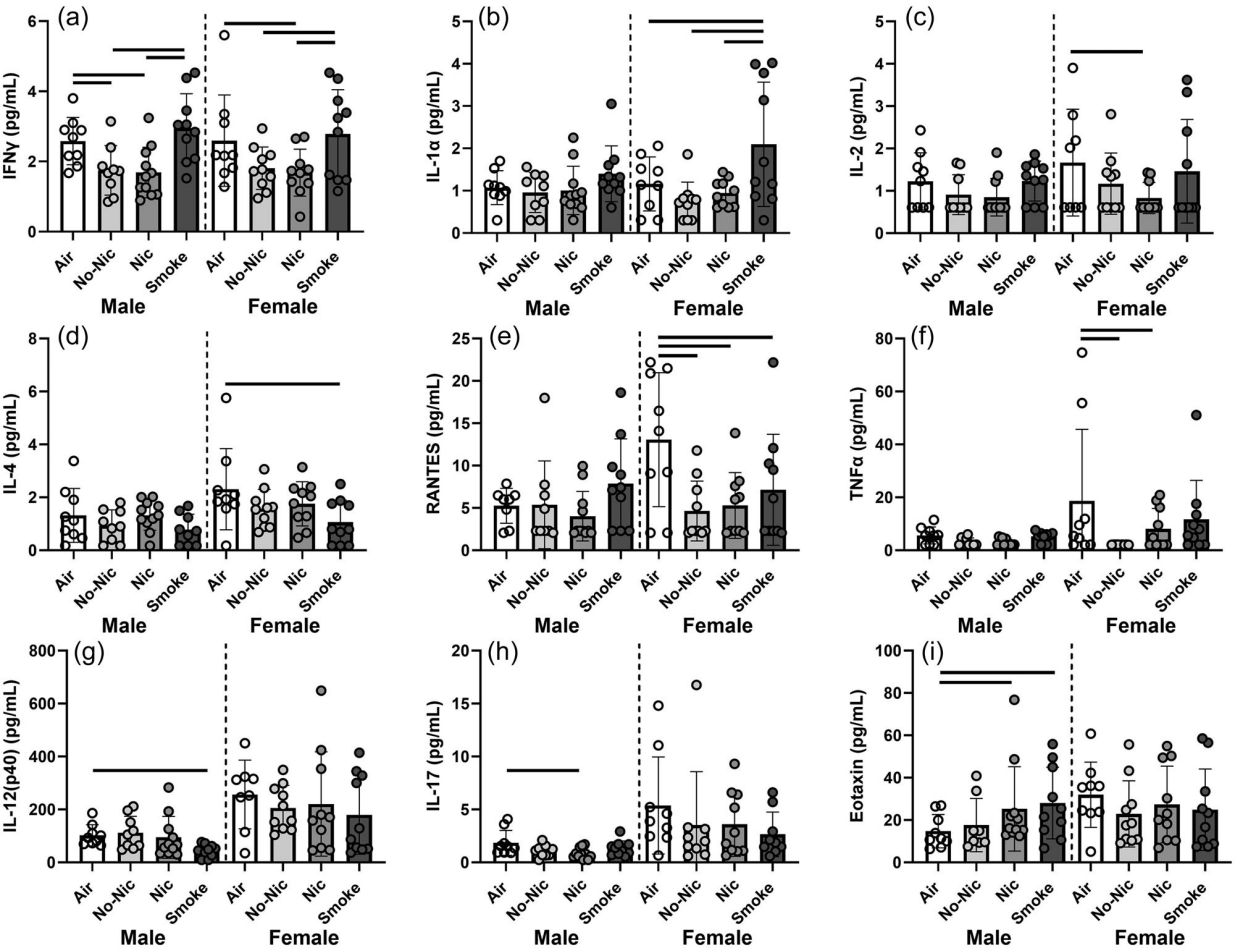
Bronchoalveolar lavage mediator levels for male and female BALB/c mice with house dust mite‐induced allergic airways disease and exposed to Air, e‐cigarette aerosol with and without nicotine (Nic and No‐Nic, respectively) or cigarette smoke (Smoke). Note different scales. Circles represent individual mice (*n* = 11–12 per sex per treatment). Bars connect treatments that are significantly different from each other (*P* < 0.05).

BAL interferon γ (IFNγ) levels were significantly impacted by treatment for both sexes (Figure 3a). For female mice, those exposed to Smoke had significantly more IFNγ than either e‐cigarette aerosol‐exposed treatment (*P* < 0.017 in both cases), while female Air mice had significantly higher IFNγ than female Nic mice (*P* = 0.020). For males, those exposed to Smoke, and those exposed to Air had significantly higher IFNγ than either e‐cigarette‐exposed treatment (*P* < 0.027 in all cases).

For some mediators, levels were significantly impacted by treatment in one sex of mouse only. IL‐1α, IL‐2, IL‐4, RANTES (regulated upon activation, normal T cell expressed and secreted) and tumour necrosis factor α (TNF‐α) were significantly impacted by treatment in female (but not male) mice (Figure 3). Smoke‐exposed females had significantly higher levels of IL‐1α in their BAL compared with all other treatments (*P* < 0.017 in all cases; Figure 3b). There were no other differences between treatments for this mediator (*P* < 0.07 in all cases). Female Nic mice had significantly less IL‐2 compared with Air females (*P* = 0.037; Figure 3c) and female Air mice had significantly higher IL‐4 compared with female Smoke mice (*P* = 0.009; Figure 3d). There was no effect of treatment on RANTES levels in the BAL of male mice (*P* > 0.120 in all cases); however, female Air mice had significantly higher levels of RANTES compared with all other female mouse treatments (*P* < 0.021 in all cases; Figure 3e). Similarly, there was no effect of treatment on TNF‐α levels in the BAL of male mice (*P* > 0.054 in all cases); however, female Air mice had significantly higher levels of TNF‐α compared with female NIC and female No‐Nic treatments (*P* < 0.030 in both cases; Figure 3f). Female Air mice were not significantly different from female Smoke mice for this parameter (*P* = 0.216).

IL‐12(p40), IL‐17 and eotaxin levels were only significantly impacted by treatment in male mice (Figure 3). Male Smoke mice had significantly lower IL‐12(p40) compared with all other male treatments (*P* < 0.021 in all cases; Figure 3g). There was no effect of treatment on this mediator for female mice (*P* > 0.256 in all cases). Overall, female mice had higher levels of this mediator, regardless of treatment. Similarly, females typically had higher levels of IL‐17 than males, although there was no effect of treatment on this mediator for female mice (*P* > 0.071 in all cases; Figure 3h). Male Nic mice had lower levels of IL‐17 than male Air mice (*P* = 0.037). There was no effect of treatment on eotaxin levels in the BAL of female mice (*P* > 0.280 in all cases); however, male Air mice had significantly lower levels of eotaxin compared with both male‐Nic and male Smoke mice (*P* = 0.040 and 0.021, respectively; Figure 3i).

There was no significant effect of treatment on levels of IL‐5, macrophage inflammatory protein (MIP)‐1α, MIP‐1β, keratinocyte chemoattractant (KC) or monocyte chemoattractant protein (MCP)‐1 for either males (*P* > 0.076 in all cases) or females (*P* > 0.087 in all cases). Female mice generally had higher levels of MIP‐1α and MIP‐1β than males regardless of treatment, although this pattern was not statistically significant.

### 3.8 Lung structure

There was no effect of sex or treatment on either ASM area, or epithelial thickness (*P* > 0.353 in all cases).

## 4 DISCUSSION

This study aimed to determine the effects of e‐cigarette exposure on allergic airways disease severity in male and female BALB/c mice, specifically focussing on primary respiratory outcomes including lung function, cellular inflammation and lung/airway remodelling. We employed a house dust mite‐induced allergic airways disease phenotype, generated over 7 weeks, and then superimposed e‐cigarette aerosol, cigarette smoke or air exposure for the final 2 weeks. Our goal was to assess whether e‐cigarette exposure would exacerbate key features of allergic airways disease and to compare effects with both cigarette smoking and air exposure. The majority of significant differences were seen with respect to Smoke‐exposed mice compared with Air‐exposed mice, with the e‐cigarette groups typically similar to Air controls. That said, there were complex effects of e‐cigarette exposure (with and without nicotine) on outcomes, primarily with respect to immunodysregulatory impacts (levels of key mediators in bronchoalveolar lavage), including suppression of IFNγ.

As expected in this model, the greatest impacts on functional and inflammatory outcomes were seen after cigarette smoke exposure (Tables 2 and 3, and Figure 1). E‐cigarette exposure primarily impacted immunological outcomes, including levels of key mediators in lavage (Figure 3). It is important to reiterate that all mice involved in the present study had undergone HDM treatment 5 days/week for 7 weeks, with e‐cigarette/smoke exposures only occurring for the final 2 weeks. This HDM protocol is known to elicit significant pulmonary inflammation, airway remodelling and airway hyperreactivity to methacholine in BALB/c mice (Johnson et al., 2004), which may mean that subtle effects of e‐cigarette/smoke exposure could have been overwhelmed by the effects of the HDM. A previous, similar study by Chapman et al. (2019) only treated mice seven times with HDM, and did not have a cigarette smoke‐exposed comparison group (Chapman et al., 2019). In that study, mice were exposed to either room air or e‐cigarette aerosol for 30 min twice/day, 6 days/week from day 0 to 18. Five flavours with and without nicotine (12 mg/mL), plus vehicle control were tested and complex flavour‐specific effects were seen. Similar to our study, very limited effects of e‐cigarette aerosol were measured on lung function parameters, with only one flavour of nicotine‐free e‐cigarette significantly impacting tissue elastance at the highest dose of MCh (Hogan et al., 2024). We identified effects of treatment both at FRC, and in terms of responsiveness and sensititvity to MCh – but these were largely due to cigarette smoke exposure, not e‐cigarette aerosol exposure. That said, the fact that R_aw_, G and H were higher at FRC in e‐cigarette aerosol‐exposed female mice compared with Smoke‐exposed female mice is concerning from an asthma perspective. Higher values for these parameters are indicative of increased resistance of both the main conducting and peripheral airways, in addition to higher stiffness of the lung parenchyma, an increase in parallel airway heterogeneity (Lutchen & Gillis, 1997; Lutchen et al., 1996) and potentially airway closure (Lutchen & Gillis, 1997) all of which would increase the effort required for ventilation. In this model, nicotine presence/absence had little impact for these parameters (Table 2), suggesting that other components of the e‐cigarette aerosols are responsible for the effects seen (Chun et al., 2018). This is supported by another study whereby flavour‐specific impacts on lung function in a murine model of asthma have been noted (Chapman et al., 2019). There are limited other published data in this space, although in our previous study, female mice exposed to e‐cigarette aerosols for 8 weeks had significantly higher G and H than Air‐exposed controls at FRC, regardless of nicotine content. Mice exposed to glycerin‐based e‐cigarette aerosols also were more responsive to MCh for G (Larcombe et al., 2017). The increase in G could possibly be attributed to cellular inflammation, mucus secretion or greater ASM contraction (Vardavas et al., 2012). In the present study, we did not measure any effect of treatment on responsiveness to MCh in terms of H. This is consistent with Chapman et al. (2019), which found no effect of e‐cigarette aerosol, regardless of nicotine content or flavour, on H in mice with allergic airways disease (Chapman et al., 2019). Unexpectedly, Smoke mice had lower R_aw_ than Air controls, although this was only statistically significant for males. Long‐term cigarette smoke exposure alone has previously been shown to increase airway resistance in mice (Matz et al., 2023; Zhao et al., 2024). Conversely, responsive to MCh for R_aw_ was greater for both male and female Smoke‐exposed mice (Figure 1a), which is consistent with our previous research (Larcombe et al., 2017), and human studies whereby smoking asthmatics have worse asthma control (Thomson et al., 2013).

We did not see any effects of e‐cigarette aerosol, or smoke exposure, on most cell types in the bronchoalveolar lavage (Figure 2). The effects of e‐cigarettes on pulmonary inflammation in mice are complex, with some studies showing strong inflammatory effects (Taha et al., 2020), our previous work showing no impacts (Larcombe et al., 2017), while others show anti‐inflammatory outcomes (Chapman et al., 2019). In the study by Chapman and colleagues, all flavours of e‐cigarette containing nicotine suppressed airway inflammation (but did not alter airway hyperresponsiveness or lead to airway remodelling) (Chapman et al., 2019). The suppression of inflammation after nicotine containing e‐cigarette exposure was not surprising as nicotine has known anti‐inflammatory properties (Kalra et al., 2004; Mabley et al., 2011). Nicotine has been shown to suppress inflammation caused by allergen exposure and reduce eosinophils, allergen‐specific IgE and Th_2_ cytokine levels without substantially impacting the airway epithelium in rats (Mishra et al., 2008). However, it also been shown to increase the viscosity of airway mucus (Chen et al., 2014) and increase mucus secretion by the epithelial cells (Gundavarapu et al., 2012). In the present study, it is likely that the inflammatory effects elicited by the HDM overwhelmed most additional impacts of e‐cigarette aerosol or smoke exposure. This is supported by the observation that the total cellular inflammation in Air controls was ∼350,000 to 400,000 cells per mL, which is roughly seven times greater than seen in non‐HDM‐exposed controls using our techniques (Larcombe et al., 2017). Consistent with some other studies in which the inflammatory impacts of cigarette smoke were investigated (Botelho et al., 2011; Gahring et al., 2018; Robbins et al., 2005), we also found that Smoke‐exposed groups had reduced levels of eosinophils in their bronchoalveolar lavage fluid, with the effect more apparent in male mice. Accumulation of pulmonary eosinophils is a characteristic response to exposure to allergens, including HDM (Jacquet, 2013), such that its modification in this model could be interpreted as an impaired response to HDM. This effect may be at least partially driven by nicotine, as mice exposed to aerosolised nicotine also display reduced eosinophils after HDM exposure (Gahring et al., 2020). Other studies have also shown that nicotine contains anti‐inflammatory properties (Kalra et al., 2004; Mabley et al., 2011). However, other mechanisms are also working, as we did not see similar suppression of eosinophils in our Nic treatment. Further, cellular inflammation is an important mechanism contributing to airway hyper‐responsiveness (Cockcroft & Davis, 2006). However, in the present study, the pattern of cellular inflammation did not strongly correspond to the lung function results. Such dissociation between airway inflammation and airway hyper‐responsiveness has been demonstrated before (Tournoy et al., 2000), suggesting that airway inflammation is not a main factor contributing to altered lung function in this study.

As with cellular inflammation, the effects of e‐cigarette aerosol/smoke exposure after HDM exposure on mediator levels were complex, with few clear trends (Figure 3). For 14 out of 23 mediators assessed, levels were either entirely or mostly below the limit of detection, or there was no effect of treatment. That said, some important effects were seen, particularly with respect to suppression of certain mediators by e‐cigarette exposure. The most obvious of these was IFN‐γ, which was significantly lower in e‐cigarette‐exposed mice compared with Smoke for both sexes. IFN‐γ has many functions ranging from pathogen defence to immune regulation (Schroder et al., 2004). In a similar study to ours, Song et al. (2023) found no effect of e‐cigarette exposure on levels of IFN‐γ in asthmatic mice (Song et al., 2023); however, they did not have a cigarette smoke‐exposed comparator group, and there were other methodological differences. IFN‐γ levels have previously been shown to be reduced after e‐cigarette exposure in mice (Kalininskiy et al., 2021; Sussan et al., 2015) and in *ex vivo* human samples (Hogan et al., 2024). This suppression of innate immunity has important implications for asthma development and exacerbation (Mikhail & Grayson, 2019). Similarly, in female mice, we similarly saw a suppression of IL‐1α (compared with Smoke) and of RANTES (compared with Air). IL‐1α plays an essential role in producing an inflammatory immune response (Migliorini et al., 2020). It is increased in asthma, and by cigarette smoke (Dinarello, 1996), so it is not surprising that it was highest in Smoke mice that also had an allergic airways disease phenotype. RANTES is a pro‐inflammatory cytokine released at the site of inflammation (Appay & Rowland‐Jones, 2001). It is increased in the BAL fluid of asthmatic patients (Folkard et al., 1997) and it is upregulated in mice after repeated allergen challenge (Koya et al., 2006). RANTES is also involved in the upregulation of IFN‐γ, such that the lower levels of RANTES and IFN‐γ seen in e‐cigarette‐exposed mice in this study may be correlated.

A somewhat unexpected finding of this study was no effect of e‐cigarette/smoke treatment on serum IgE levels. Such exposures have previously been shown to impair airway epithelial barrier structure and function (Ghosh et al., 2020), which has in turn been linked with asthma (Hellings & Steelant, 2020). Therefore, we expected that e‐cigarette/smoke‐exposed mice would have higher serum IgE levels than Air controls as an impaired barrier would facilitate HDM sensitisation. This was not the case, and although we did not directly assess epithelial barrier integrity, it could be assumed that any additional barrier dysfunction elicited by e‐cigarette aerosol/smoke exposure was negligible compared to that caused by the previous HDM exposure.

There are some key strengths and limitations to this study. A key strength is the study of both male and female mice, which allowed for the differential effects of sex to be assessed. Additionally, compared with similar studies, we provide a more comprehensive suite of outcome measures (including lung function and a wide range of immunological outcomes). An important limitation, however, is the dose/duration of both the HDM and smoke/e‐cigarette exposures. Seven weeks of HDM exposure was potentially excessive and it may have overwhelmed more subtle effects of e‐cigarette or smoke exposures. We, and others, have previously subjected mice to considerably shorter HDM exposure protocols (Phan et al., 2014). A longer exposure duration was required in this study as we aimed to establish an allergic airways disease phenotype prior to e‐cigarette/smoke exposure.

### 4.1 Conclusion

In summary, this study shows that e‐cigarette exposure (with and without nicotine) had minimal significant impact on asthma severity in mice, although it did lead to immune system dysregulation. There were trends towards impaired lung function at FRC and sensitivity to methacholine, but statistically significant differences in these parameters were not observed, possibly due to the HDM exposure regime. Measured effects were slightly more pronounced in female mice, which may be due to their smaller size. While there are still considerable gaps in knowledge with respect to the effects of e‐cigarettes on asthma, our findings may be instrumental in informing the public and policymakers on the harms of e‐cigarette use in a susceptible population.

## AUTHOR CONTRIBUTIONS

**Jenield J. D'abreo**: Conceptualisation, formal analysis, investigation, methodology, writing—original draft, writing—review and editing. **Emily K. Chivers**: Formal analysis, investigation, methodology, supervision, writing—review and editing. **Peter B. Noble**: Methodology, supervision, writing—review and editing. **Luke J. Berry**: Formal analysis, methodology, writing—review and editing. Rachel R. Huxley: conceptualisation, funding acquisition, writing—review and editing. **Peter J. Franklin**: Conceptualisation, conceptualisation, funding acquisition, writing—review and editing. **Benjamin J. Mullins**: Conceptualisation, funding acquisition, writing—review and editing. **Katherine R. Landwehr**: Formal analysis, investigation, methodology, writing—review and editing. **Alexander N. Larcombe**: Conceptualisation, data curation, formal analysis, funding acquisition, investigation, methodology, project administration, resources, supervision, writing—review and editing. All authors have read and approved the final version of this manuscript and agree to be accountable for all aspects of the work in ensuring that questions related to the accuracy or integrity of any part of the work are appropriately investigated and resolved. All persons designated as authors qualify for authorship, and all those who qualify for authorship are listed.

## CONFLICT OF INTEREST

The authors declare that they have no known competing financial interests or personal relationships that could have appeared to influence the work reported in this paper.

## Data Availability

The datasets used and analysed during the current study are available from the corresponding author on reasonable request.
